# Indoor air quality and health in schools*

**DOI:** 10.1590/S1806-37132014000300009

**Published:** 2014

**Authors:** Ana Maria da Conceição Ferreira, Massano Cardoso

**Affiliations:** Scientific Committee, Environmental Health Course, Coimbra School of Health Technology, Coimbra, Portugal; University of Coimbra School of Medicine, Coimbra, Portugal

**Keywords:** Air pollution, indoor, Signs and symptoms, respiratory

## Abstract

**Objective:**

To determine whether indoor air quality in schools is associated with the prevalence of allergic and respiratory diseases in children.

**Methods:**

We evaluated 1,019 students at 51 elementary schools in the city of Coimbra, Portugal. We applied a questionnaire that included questions regarding the demographic, social, and behavioral characteristics of students, as well as the presence of smoking in the family. We also evaluated the indoor air quality in the schools.

**Results:**

In the indoor air of the schools evaluated, we identified mean concentrations of carbon dioxide (CO_2_) above the maximum reference value, especially during the fall and winter. The CO_2_ concentration was sometimes as high as 1,942 ppm, implying a considerable health risk for the children. The most prevalent symptoms and respiratory diseases identified in the children were sneezing, rales, wheezing, rhinitis, and asthma. Other signs and symptoms, such as poor concentration, cough, headache, and irritation of mucous membranes, were identified. Lack of concentration was associated with CO_2_ concentrations above the maximum recommended level in indoor air (p = 0.002). There were no other significant associations.

**Conclusions:**

Most of the schools evaluated presented with reasonable air quality and thermal comfort. However, the concentrations of various pollutants, especially CO_2_, suggest the need for corrective interventions, such as reducing air pollutant sources and improving ventilation. There was a statistically significant association between lack of concentration in the children and exposure to high levels of CO_2_. The overall low level of pollution in the city of Coimbra might explain the lack of other significant associations.

## Introduction

People spend, on average, over 80% of their time in buildings, being therefore exposed to higher concentrations of pollutants indoors than outdoors. Children are vulnerable to such exposure, being at an increased risk of developing respiratory diseases, such as asthma.^(^ ^1^ ^,^ ^2^ ^)^ Asthma is the leading cause of hospitalization and school absenteeism, negatively affecting learning and academic performance in students in Western countries.^(^ ^3^ ^,^ ^4^ ^)^

Numerous strategies can be implemented in order to reduce the risk of exposure to pollutants; good indoor air quality is indispensable and is achieved through appropriate room ventilation, as well as through ventilation and exhaust of combustion fumes and gases. Temperature control and humidity control are also indispensable. Other, practical, recommendations include daily breathing exercises and outdoor leisure activities.^(^ ^5^ ^)^

Given that children spend a long time in school buildings, we can predict that the conditions in such buildings affect the incidence of respiratory symptoms.^(^ ^6^ ^,^ ^7^ ^)^ Several studies involving children have shown a positive association between exposure to air pollutants and increased morbidity and mortality from respiratory problems.^(^ ^8^ ^-^ ^10^ ^)^

The objective of the present study was to determine whether indoor air quality in elementary schools in the city of Coimbra, Portugal, is associated with the prevalence of allergic and respiratory diseases in children.

## Methods

The study focused on public and private elementary (1st-4th grade) schools in the Municipality of Coimbra. The schools were selected on the basis of a comparative analysis of the 81 schools and 230 classrooms (the network of public and private elementary schools) in the Municipality of Coimbra, the 2008/2015 Education Charter of the Municipality of Coimbra being taken into consideration. Various generic, demographic, and social indicators were used in the analysis. When there was only one elementary school in a given parish, that school was necessarily chosen so that all of the parishes of the Municipality of Coimbra were represented. Other aspects were taken into consideration, including school size (larger schools being selected), school surroundings, human activity, nearby traffic, and industrial activity in the area. A non-probabilistic convenience sampling procedure was used in order to select the sample. The inclusion criteria were as follows: selection of at least one school per parish; use of the aforementioned comparison criteria; and authorization from the Direção Regional de Educação do Centro (DREC, Central Regional Education Board), school clusters, and school principals. The sample consisted of 51 schools, which corresponded to 81 classrooms (35 1st-grade classrooms, 34 4th-grade classrooms, and 12 mixed classrooms). Of the total of schools, 32 were located in predominantly urban parishes, 17 were located in moderately urban parishes, and 2 were located in predominantly rural parishes.^(^ ^11^ ^)^

Indoor air quality was evaluated in the fall/winter and spring/summer. In order to characterize indoor air quality, we measured temperature, relative humidity (RH), and the concentrations of the following: carbon monoxide (CO); carbon dioxide (CO_2_); ozone (O_3_); nitrogen dioxide; sulfur dioxide (SO_2_); volatile organic compounds (VOCs); formaldehyde; particulate matter of 2.5 µm in diameter (PM2.5); and PM of 10 µm in diameter (PM10). The aforementioned measurements were performed in the fall/winter (between November of 2010 and February of 2011) and in the spring/summer (between March of 2011 and June of 2011). 

According to Portuguese National technical standards NT-SCE-02,^(^ ^12^ ^)^ pollutants should be measured in the representative period of activity, either 2-3 h after the initiation of activities or after equilibrium conditions have been reached. All measurements of indoor air quality were performed during regular classes, i.e., within approximately 2 h after the beginning of classes (in the morning or in the afternoon), by placing the equipment in the most central position in each classroom and at the level of the airways of the students in the sitting position. All measurements were performed in accordance with Portuguese National technical standards NT-SCE-02,^(^ ^12^ ^)^ at 1 m from the floor and at least 3 m from the walls, between 10:30 a.m. and 5:30 p.m., for a period of 30 min, samples for the measurement of PM, VOCs, and the remaining parameters of indoor air quality being collected every 30 s, every 15 s, and every 60 s, respectively. All measurements were performed over the course of one week. On average, two measurements per day were performed in each classroom, depending on the size of the classroom. 

Outdoor air quality was measured during recess, the measurements being performed at 1 m from the ground and at least 1 m from the external walls of the schools studied.^(^ ^13^ ^)^

For real-time measurement of air quality parameters, the following portable devices were used: VelociCalc 9555-P (TSI Inc., Shoreview, MN, USA), in order to measure temperature, RH, and the concentrations of CO and CO_2_; series 500 handheld monitor (Aeroqual Ltd., Auckland, New Zealand), in order to measure the concentration of O_3_; QRAE (ERA Systems Europe ApS, Kastrup, Denmark), in order to measure the concentrations of nitrogen dioxide and SO_2_; Formaldemeter htV (PPM Technology, Caernarfon, UK), in order to measure formaldehyde; Voyager (Photovac Inc., Waltham, MA, USA), in order to measure the concentrations of VOCs; and DUSTTRACK (TSI Inc.), in order to measure the concentration of PM. The devices were calibrated before sampling, the "blank" (or zero) standard being used whenever necessary in order to compare the results obtained in cases of measurements performed after changing sensors. We took into consideration the conversion of the readings on the basis of the variations in temperature and pressure. 

Information on the students was collected by a questionnaire that resulted from different pre-tests. Those pre-tests focused on the time it took parents or legal guardians to complete the questionnaire, as well as on their understanding of the questions depending on the topics covered. The final version covered the following topics: family characteristics (nuclear family, single-parent family, or extended family); housing characteristics (place of residence, mean length of stay, type of housing, and thermal conditions, among others); and information regarding symptoms/diseases and physical activity levels in the children studied. Envelopes containing the questionnaires were delivered to the teachers by the principal investigator, and the teachers instructed the students to deliver the questionnaires to their parents/legal guardians. 

A non-probabilistic convenience sampling procedure was used in order to select elementary school children in the 1st-4th grade. Of a total of 4,319 children, 1,019 were selected. 

We measured the weight and height of the children and subsequently calculated the body mass index, by dividing the weight (in kg) by height (in m^2)^. Children with a body mass index above the 95th percentile were classified as overweight on the basis of the 2000 US Centers for Disease Control and Prevention percentile distribution for gender and age.^(^ ^14^ ^)^

The results are presented by school, and the proportions were compared by the chi-square test and Fisher's exact test. For comparison of continuous variables, ANOVA or its non-parametric equivalent (i.e., the Kruskal-Wallis test) was used. The relative risk was estimated by calculating ORs and their 95% CIs. All statistical analyses were performed with the IBM SPSS Statistics software package, version 19.0 (IBM Corporation, Armonk, NY, USA). 

In the present study, we strategically divided the schools into two categories, on the basis of CO_2_ levels: no health risk (i.e., mean CO_2_ concentrations below the maximum reference value established by Portuguese government Decree-Law no. 79/2006, i.e., ≤ 984 ppm); and health risk (i.e., mean CO_2_ concentrations > 984 ppm).

## Results

The mean age of 1st-grade students was 6.20 ± 0.42 years, and the mean age of 4th-grade students was 9.25 ± 0.48 years. Most (51.63%) of the children were male. There was a relatively uniform distribution of the 493 female children between the two grades studied. A similar trend was observed in male students. 

By measuring weight and height, we found that 5% of the children included in the study were obese. 

We also found that 84.6% of the children practiced sports. In 7 of the schools studied, 100% of the children practiced sports. 

Regarding the level of education of the parents/legal guardians (by area of residence, i.e., parish), of the 1,014 respondents, 436 (43%) had finished college, and 48 (4.7%) had had only 4 years of schooling. Most of the parents/legal guardians resided in predominantly urban and moderately urban parishes (61% and 26%, respectively). 

Regarding the age of the households where the children lived, we found that there were statistically significant differences: 71.3% of the households were less than 21 years of age, and 8.2% were over 40 years of age. In addition, 25.4% of the households had mold, and there were significant differences among the households in terms of the presence of moisture and a heating system (53.7% of the children having been found to live in households without heating systems). 

The proportion of students living in households with heating and fewer signs of moisture was higher in the schools whose students had parents/legal guardians who had a higher level of education. 

Table 1 shows the mean concentrations of indoor air quality parameters in the elementary schools in the two sampling periods, i.e., fall/winter and spring/summer.

**Table 1:** - Distribution of the concentrations of pollutants in the indoor air of the 81 classrooms studied, by season.a

Pollutants	Sampling period		Δfall/winter – spring/summer (mean)	Maximum reference value according to Portuguese law
	Fall/winter	Spring/summer		
CO, ppm	0.42 ± 0.53*	0.14 ± 0.13	0.28	10.7
CO_2_, ppm	1578.16* ± 12.49	1152.80 ± 595.41	425.36	984
PM2.5, mg/m^3^	0.08 ± 0.04	0.10 ± 0.03	−0.02	Not mentioned
PM10, mg/m^3^	0.12 ± 0.05	0.11 ± 0.03	0.006	0.15
O3, ppm	0.002 ± 0.060	0.0009 ± 0.0040	0.001	0.10
VOCs, ppb	97.82 ± 73.72	90.51 ± 65.66	7.31	260
SO_2_, ppm	0.005 ± 0.020	0.004 ± 0.030	0.001	Not mentioned
Formaldehyde, ppm	0.01 ± 0.01*	0.02 ± 0.02	−0.01	0.08

CO : carbon monoxide

CO2 : carbon dioxide

PM2.5 : particulate matter of 2.5 µm in diameter

PM10 : particulate matter of 10 µm in diameter

O3 : ozone

VOCs : volatile organic compounds

SO2 : sulfur dioxide

a Values expressed as mean ± SD

*p < 0.0001 (Student's t-test for paired samples or Wilcoxon t-test)

Mean concentrations of CO and CO_2_ were significantly higher in the fall/winter than in the spring/summer (p < 0.001). There was a reduction of 0.28 ppm in the concentration of CO from one sampling period to another. Regarding CO_2_ levels, there was a reduction of 425.36 ppm in the spring/summer. We found mean CO_2_ concentrations that were well above the maximum reference value (i.e., 984 ppm) and therefore posed health risks to the children studying in those schools. 

There were significant differences between the mean formaldehyde concentration measured in the fall/winter and that measured in the spring/summer. Mean formaldehyde concentrations were found to be significantly higher in the spring/summer than in the fall/winter, formaldehyde levels having increased by 0.0103 ppm. Although there were no significant differences between the two sampling periods in terms of the remaining parameters, the concentrations of PM10, O_3_, VOCs, and SO_2_ were found to be lower in the spring/summer than in the fall/winter. Conversely, the concentration of PM2.5 was found to be higher, although not significantly so. 

In all but 2 classrooms, mean air temperatures in the fall/winter were found to be well below the reference value. In general, mean air temperatures in the spring/summer were found to be above the reference value, which was due to the external temperature and the fact that the classrooms had no cooling system. 

In both sampling periods, RH values were found to be between the lower and upper limits (30-70%). However, in the fall/winter period, 7 schools had RH values above 70%. 

Regarding the concentration of air pollutants in the outdoor air of the schools in the two sampling periods, mean concentrations of CO, CO_2_, PM2.5, PM10, and formaldehyde varied significantly. CO and CO_2_ levels were found to be significantly lower in the fall/winter than in the spring/summer. Conversely, PM2.5, PM10, and formaldehyde levels were found to be significantly higher in the spring/summer than in the fall/winter. 

Table 2 shows the most prevalent symptoms and diseases in the children studied.

**Table 2:** - Signs, symptoms, and diseases in 1st- and 4th-grade children in the city of Coimbra, Portugal.

Signs, symptoms, and diseases		School grade						Total	
		1st grade			4th grade				
		n	% column	% line	n	% column	% line	n	% column
Asthma	No	451	89.3	50.2	448	87.2	49.8	899	88.2
	Yes	54	10.7	45.0	66	12.8	55.0	120	11.8
	Total	505	100.0	49.6	514	100.0	50.4	1.019	100.0
Chronic bronchitis	No	495	98.0	49.6	502	97.7	50.4	997	97.8
	Yes	10	2.0	45.5	12	2.3	54.5	22	2.2
	Total	505	100.0	49.6	514	100.0	50.4	1.019	100.0
Rales/wheezing	No	420	83.2	48.6	444	86.4	51.4	864	84.8
	Yes	85	16.8	54.8	70	13.6	45.2	155	15.2
	Total	505	100.0	49.6	514	100.0	50.4	1.019	100.0
Sneezing attacks	No	382	75.6	50.3	377	73.3	49.7	759	74.5
	Yes	123	24.4	47.3	137	26.7	52.7	260	25.5
	Total	505	100.0	49.6	514	100.0	50.4	1.019	100.0
Allergic rhinitis	No	426	84.4	51.0	409	79.6	49.0	835	81.9
	Yes	79	15.6	42.9	105	20.4	57.1	184	18.1
	Total	505	100.0	49.6	514	100.0	50.4	1019	100.0
Breathing difficulties	No	459	90.9	50.1	457	88.9	49.9	916	89.9
	Yes	46	9.1	44.7	57	11.1	55.3	103	10.1
	Total	505	100.0	49.6	514	100.0	50.4	1.019	100.0
Stress	No	499	98.8	49.9	502	97.7	50.1	1001	98.2
	Yes	6	1.2	33.3	12	2.3	66.7	18	1.8
	Total	505	100.0	49.6	514	100.0	50.4	1.019	100.0
Dizziness	No	497	98.4	49.7	502	97.7	50.3	999	98.0
	Yes	8	1.6	40.0	12	2.3	60.0	20	2.0
	Total	505	100.0	49.6	514	100.0	50.4	1019	100.0
Irritability	No	487	96.4	49.9	489	95.1	50.1	976	95.8
	Yes	18	3.6	41.9	25	4.9	58.1	43	4.2
	Total	505	100.0	49.6	514	100.0	50.4	1.019	100.0
Headache	No	472	93.5	50.4	465	90.5	49.6	937	92.0
	Yes	33	6.5	40.2	49	9.5	59.8	82	8.0
	Total	505	100.0	49.6	514	100.0	50.4	1019	100.0
Conjunctival irritation	No	483	95.6	49.8	486	94.6	50.2	969	95.1
	Yes	22	4.4	44.0	28	5.4	56.0	50	4.9
	Total	505	100.0	49.6	514	100.0	50.4	1.019	100.0
Insomnia	No	486	96.2	49.7	492	95.7	50.3	978	96.0
	Yes	19	3.8	46.3	22	4.3	53.7	41	4.0
	Total	505	100.0	49.6	514	100.0	50.4	1019	100.0
Cough	No	421	83.4	49.2	434	84.4	50.8	855	83.9
	Yes	84	16.6	51.2	80	15.6	48.8	164	16.1
	Total	505	100.0	49.6	514	100.0	50.4	1.019	100.0
Lack of concentration	No	402	79.6	50.8	390	75.9	49.2	792	77.7
	Yes	103	20.4	45.4	124	24.1	54.6	227	22.3
	Total	505	100.0	49.6	514	100.0	50.4	1.019	100.0

The most prevalent symptoms/diseases in the 1st-grade children were as follows: sneezing attacks, in 24%; lack of concentration, in 20%; rales and wheezing, in 17%; cough, in 16%; and allergic rhinitis, in 16%. In the 4th-grade children, the most prevalent symptoms/diseases were as follows: sneezing attacks, in 27%; lack of concentration, in 24%; allergic rhinitis, in 20%; and cough, in 16%. When we compared the children who were in the 1st grade with those who were in the 4th grade in terms of the prevalence of each symptom, we found that rales and wheezing were more common in the 1st-grade children (having been found in 55%), as was cough (in 51%). The remaining symptoms/diseases were found to be more common in the children who were in the 4th grade. 

Of all environmental parameters analyzed, CO_2_ levels showed the worst results, posing serious health risks. In the indoor air of the schools evaluated, mean CO_2_ concentrations were in general well above the maximum reference value (984 ppm), being sometimes as high as 1,942 ppm. Given that CO_2_ concentrations in indoor air were found to be much higher in the fall/winter than in the spring/summer, we sought to estimate the risk of symptoms/diseases in the elementary school children. The classrooms were classified as posing health risks or as posing no health risks on the basis of the reference value. The symptoms/diseases were reported by the parents/legal guardians through the questionnaire (Table 3).

**Table 3:** - Estimation of the risk of respiratory symptoms and diseases in the children who, during the fall/winter, were exposed to a mean carbon dioxide concentration that was above or below the maximum reference value.

Respiratory symptoms and diseases	Maximum reference value for carbon dioxide concentration							c2	df	p	OR	95% CI
	Above			Below		Total						
	n		%	n	%	n	%					
Asthma	Yes	100	11.7	20	12.3	120	11.8	0.046	1	0.831	0.946	0.567-1.579
	No	756	88.3	143	87.7	899	88.2					
	Total	856	100.0	163	100.0	1019	100.0					
Chronic bronchitis	Yes	18	2.1	4	2.5	22	2.2	0.080	1	0.777	0.854	0.285-2.556
	No	838	97.9	159	97.5	997	97.8					
	Total	856	100.0	163	100.0	1019	100.0					
Rales/wheezing	Yes	133	15.5	22	13.5	155	15.2	0.442	1	0.506	1.179	0.725-1.917
	No	723	84.5	141	86.5	864	84.8					
	Total	856	100.0	163	100.0	1019	100.0					
Sneezing attacks	Yes	223	26.1	37	22.7	260	25.5	0.810	1	0.368	1.200	0.807-1.784
	No	633	73.9	126	77.3	759	74.5					
	Total	856	100.0	163	100.0	1019	100.0					
Allergic rhinitis	Yes	160	18.7	24	14.7	184	18.1	1.457	1	0.227	1.331	0.835-2.122
	No	696	81.3	139	85.3	835	81.9					
	Total	856	100.0	163	100.0	1019	100.0					
Cough	Yes	141	16.5	23	14.1	164	16.1	0.565	1	0.452	1.200	0.745-1.933
	No	715	83.5	140	85.9	855	83.9					
	Total	856	100.0	163	100.0	1019	100.0					
Breathing difficulties	Yes	85	9.9	18	11.0	103	10.1	0.187	1	0.666	0.888	0.518-1.522
	No	771	90.1	145	89.0	916	89.9					
	Total	856	100.0	163	100.0	1019	100.0					

df : degrees of freedom

We found no significant association between the presence/absence of asthma and exposure to classrooms with/without health risks (p = 0.831). However, the prevalence of asthma was 11.8% in the total population of children studied. 

Chronic bronchitis occurred in 22 children (2.2%); however, we found no significant association between the disease and exposure to high CO_2_ levels in the classrooms during the fall/winter (p > 0.05). Rales/wheezing were reported in 155 children (prevalence, 15.2%), with no significant associations (p > 0.05). Although we found no association between sneezing attacks and exposure to high CO_2_ levels (p > 0.05), we found that, of the 856 children studying in classrooms with high CO_2_ levels (health risk), 223 (26.1%) had sneezing attacks. 

We found no association of allergic rhinitis, cough, or breathing difficulties with exposure to classrooms with or without health risks because of CO_2_ levels (p > 0.05); however, of the total of children controlled for each symptom, 184 (18.9%) had rhinitis, 164 (16.1%) had cough, and 103 (10.1%) had breathing difficulties. 

We sought to understand the distribution of non-respiratory symptoms by classroom with or without health risks during the fall/winter. The classification into presence or absence of health risks followed the methods described in the previous analysis (Table 4).

**Table 4:** - Estimation of the risk of non-respiratory signs and symptoms in the children who, during the fall/winter, were exposed to a mean carbon dioxide concentration that was above or below the maximum reference value.

Signs and symptoms	Maximum reference value for carbon dioxide concentration							c2	df	p	OR	95% CI
	Above			Below		Total						
	n		%	n	%	n	%					
Stress	Yes	14	1.6	4	2.5	18	1.8	0.529	1	0.467	0.661	0.215-2.034
	No	842	98.4	159	97.5	1001	98.2					
	Total	856	100.0	163	100.0	1019	100.0					
Dizziness	Yes	17	2.0	3	1.8	20	2.0	0.015	1	0.902	1.081	0.313-3.730
	No	839	98.0	160	98.2	999	98.0					
	Total	856	100.0	163	100.0	1019	100.0					
Irritability	Yes	40	4.7	3	1.8	43	4.2	2.718	1	0.099	2.614	0.799-8.554
	No	816	95.3	160	98.2	976	95.8					
	Total	856	100.0	163	100.0	1019	100.0					
Headache	Yes	70	8.2	12	7.4	82	8.0	0.123	1	0.726	1.121	0.593-2.118
	No	786	91.8	151	92.6	937	92.0					
	Total	856	100.0	163	100.0	1019	100.0					
Mucosal irritation	Yes	42	4.9	8	4.9	50	4.9	0.0001	1	0.999	1.000	0.460-2.171
	No	814	95.1	155	95.1	969	95.1					
	Total	856	100.0	163	100.0	1019	100.0					
Insomnia	Yes	33	3.9	8	4.9	41	4.0	0.393	1	0.531	0.777	0.352-1.714
	No	823	96.1	155	95.1	978	96.0					
	Total	856	100.0	163	100.0	1019	100.0					
Lack of concentration	Yes	206	24.1	21	12.9	227	22.3	9.888	1	0.002	2.143	1.320-3.478
	No	650	75.9	142	87.1	792	77.7					
	Total	856	100.0	163	100.0	1019	100.0					

df : degrees of freedom

On the basis of the reports by the parents/legal guardians of the 1,019 children included in the present study, we calculated the prevalence of the following signs and symptoms: stress, 1.8%; dizziness, 2.0%; irritability, 4.2%; headache, 8%; mucosal irritation, 4.9%; and insomnia, 4.0%. None of the parameters evaluated were found to be significantly associated with the presence or absence of health risks in the classrooms (p > 0.05). Lack of concentration was found to be associated with exposure to indoor air in which CO_2_ levels were > 984 ppm (p = 0.002). The probability of having poor concentration was 2.143 times higher in the children who were exposed to CO_2_ levels > 984 ppm than in those who were not. Of the total of children investigated in the present study, 227 (22.3%) were found to have poor concentration. 

We sought to determine whether asthma was associated with household exposure to tobacco smoke (Table 5). We found that 361 (35.43%) of the parents/legal guardians were smokers, and, of those, 252 (69.8%) had the habit of smoking in the household. Although we found no association between tobacco exposure and asthma (p > 0.05), 30 (11.9%) of the 252 children exposed to tobacco smoke had asthma.

**Table 5:** - Association between asthma in children and smoking parents/legal guardians (N = 361) smoking at home.

Parents/legal guardians smoking at home	Asthma in children			p	OR	95% CI
	Yes	No	TOTAL			
Yes	30 (11.9)	222 (88.1)	252 (100.0)	0.305	1.502	0.687-3.280
No	9 (8.3)	100 (91.7)	109 (100.0)			
TOTAL	39 (10.8)	322 (89.2)	361 (100.0)			

## Discussion

Children constitute a risk group and are vulnerable to poor indoor environmental quality. The development of respiratory diseases is associated with poor air quality in school buildings.^(^ ^6^ ^,^ ^15^ ^)^

In the present study, the concentrations of the pollutants analyzed were in general below the maximum reference value, the exception being the concentration of CO_2_. However, we found significant concentrations of certain parameters, namely PM10 and VOCs. 

The results of the present study showed inadequate classroom air renewal. Because of the total volume of the classrooms, the total number of classroom occupants, and the climatic conditions, classroom ventilation during breaks is insufficient to reduce CO_2_ levels to acceptable levels. Several recent studies, some of which were conducted in Portugal,^(^ ^2^ ^,^ ^16^ ^,^ ^17^ ^)^ showed high CO_2_ levels in schools as a result of high occupancy and inadequate ventilation.^(^ ^18^ ^-^ ^21^ ^) ^These results raise several questions to be answered by governments and those responsible for this area, especially after the latest restructuring carried out at the level of schools and school clusters. Large clusters increase the number of students per classroom and, consequently, reduce the number of classes, leading us to ask the following question: Won't this reduce indoor air quality and therefore have a negative impact on the health of children? 

In the present study, the most prevalent symptoms/diseases were sneezing attacks, lack of concentration, allergic rhinitis, cough, rales/wheezing, and asthma. Other studies have reported similar results.^(^ ^6^ ^,^ ^22^ ^,^ ^23^ ^)^ In addition, lack of concentration was associated with CO_2_ levels > 984 ppm in indoor air (p = 0.002). The probability of having poor concentration was 2.143 times higher in the children who were exposed to CO_2_ levels above the reference range than in those who were not. In one study, high CO_2_ levels in schools were associated with rales and cough in children.^(^ ^24^ ^)^

Exposure to tobacco smoke in indoor environments results in an increased risk for bronchitis and asthma, among others.^(^ ^25^ ^,^ ^26^ ^)^ Of the 361 parents/legal guardians who were smokers, 30.2% did not smoke at home and 69.8% did. We therefore sought to determine whether there was a relationship between parents who smoked at home and symptoms/diseases in the children. We found that most of the parents/legal guardians who smoked at home had children with asthma (76.9%), chronic bronchitis, rales/wheezing (69.0%), sneezing attacks (56.0%), allergic rhinitis (65.0%), stress (66.7%), dizziness (85.7%), irritability (71.4%), headache (75.0%), irritation of the mucous membranes of the eyes (66.7%), dry cough (53%), insomnia (72.7%), breathing difficulties (70.5%), and lack of concentration (62.2%). The health effects of passive exposure to tobacco smoke have been the subject of numerous investigations. It is known that children are particularly susceptible, being at an increased risk of developing allergic airway disease, particularly bronchial asthma, and the diseases is more severe in children.^(^ ^27^ ^,^ ^28^ ^)^ Therefore-and on the basis of our results, which are worrisome from an environmental standpoint in schools-it is desirable that children be exposed to lower levels of all contaminants at home, including tobacco smoke contaminants. 

Nowadays, people spend most of their time in enclosed spaces, such as school buildings. Poor indoor air quality in such buildings is associated with the development of respiratory diseases. In the present study, the most prevalent symptoms were sneezing attacks and lack of concentration. 

Most of the schools studied had reasonable air quality and thermal comfort. However, the concentrations of various pollutants, especially CO_2_, suggest the need for corrective interventions, such as reducing air pollutant sources and improving ventilation. Several studies have shown high CO_2_ levels in schools as a result of high occupancy or inadequate ventilation.^(^ ^2^ ^,^ ^18^ ^-^ ^21^ ^)^ We found a statistically significant association between poor concentration and exposure to high CO_2_ levels. One possible explanation for the lack of other significant associations is the overall low level of pollution in the city of Coimbra. 

Potential limitations of the present study include the fact that the information regarding symptoms/diseases in the children was reported by their parents/legal guardians. The perception that parents/legal guardians have of their children might not correspond to reality. 

The present study allowed us to assess the risks to which the population is exposed and provide guidelines for the development of measures to minimize these risks. We hope that our findings will contribute to environmental health planning in school buildings and the improvement of political strategies to promote quality of life. 
