# Supplementary material for: Indoor air quality and health in schools
Source: J Bras Pneumol. 2014 May-Jun;40(3):259–68. doi: 10.1590/S1806-37132014000300009 (PMC4109198; doi:10.1590/S1806-37132014000300009)
Supplement: Translation

# Translation: Qualidade do ar interno e saúde em escolas*

**Authors:** Ana Maria da Conceição Ferreira, Massano Cardoso

**Affiliations:** Comissão Cientifica do Curso de Saúde Ambiental, Escola Superior de Tecnologia da Saúde de Coimbra, Coimbra, Portugal; Faculdade de Medicina, Universidade de Coimbra, Coimbra, Portugal

**Keywords:** Poluição do ar em ambientes fechados, Bem-estar da criança, Sinais e sintomas respiratórios

## Abstract

**Objetivo:**

Determinar se há uma associação entre a qualidade do ar interno em escolas e a prevalência de patologias alérgicas e respiratórias nas crianças que as frequentam.

**Métodos:**

Foram avaliados 1.019 alunos de 51 escolas de ensino básico na cidade de Coimbra, Portugal. A avaliação foi realizada através de um questionário com questões referentes a características demográficas, sociais e comportamentais dos alunos, assim como presença de hábitos tabágicos na família. Foi ainda avaliada a qualidade do ar interno nas escolas.

**Resultados:**

Foram identificadas concentrações médias de dióxido de carbono (CO_2_) no interior das salas de aula acima da concentração máxima de referência, principalmente no período de outono/inverno, chegando a valores de 1.942 ppm, o que implica elevado risco potencial para a saúde das crianças. Os sintomas/patologias respiratórias mais prevalentes nas crianças foram crises de espirros, rinite alérgica, estertores/sibilos e asma. Outros sinais e sintomas verificados foram falta de concentração, tosse, dores de cabeça e irritação das mucosas. A falta de concentração das crianças foi associada ao ar interno das salas de aula com valores acima do máximo recomendado para CO_2_ (p = 0,002). Não houve outras associações significativas.

**Conclusões:**

A maioria das escolas estudadas apresentava razoável qualidade do ar e conforto térmico, embora a concentração de vários poluentes, sobretudo CO_2_, sugere a necessidade de intervenções corretivas, como redução de fontes emissoras de poluentes e melhorias da ventilação. Houve uma associação estatisticamente significativa entre a falta de concentração nas crianças e exposição a valores elevados de CO_2_. O baixo nível de poluição na cidade de Coimbra pode explicar a falta de outras associações significativas.

## Introdução

As pessoas passam, em média, mais de 80% do seu tempo em edifícios, o que as levam a ficar expostas a maiores concentrações de poluentes no ambiente interno do que as presentes no ambiente externo. As crianças constituem um grupo de risco vulnerável, podendo surgir um aumento de doenças respiratórias, tal como a asma.(1,2) A asma é a principal causa de hospitalização e de absentismo escolar, afetando negativamente a aprendizagem e o desempenho dos alunos em países ocidentais.(3,4)

São inúmeras as estratégias que podem ser implementadas para a diminuição do risco de exposição a fatores poluentes, sendo fundamental o cuidado com a boa qualidade do ar interno nos edifícios, através de condições de arejamento adequadas, assim como de ventilação e exaustão de fumos e gases oriundos de combustão. O controle da temperatura e da umidade é igualmente indispensável. São também muitos os conselhos práticos que podem ser referenciados, tais como realizar diariamente exercícios respiratórios e privilegiar atividades de lazer ao ar livre, entre muitas outras iniciativas.(5)

As crianças permanecem na escola muito tempo, podendo-se prever que as condições existentes naqueles edifícios afetam a incidência de sintomas respiratórios.(6,7) Vários estudos com crianças demonstram uma associação positiva entre a exposição a poluentes do ar com aumento da morbidade e mortalidade devido a problemas respiratórios.(8-10)

O objetivo do presente estudo foi avaliar a associação entre a qualidade do ar do ambiente interno em escolas do primeiro ciclo do ensino básico (EB1) na cidade de Coimbra, Portugal, e a prevalência de patologias alérgicas e respiratórias na população estudantil.

Métodos

O estudo incidiu sobre escolas EB1 pertencentes a todas as freguesias do Concelho de Coimbra, independentemente de serem de natureza pública ou privada. Essas escolas foram selecionadas a partir de uma análise comparativa entre o universo das 81 escolas e das 230 salas de aula (rede do primeiro ciclo de ensino básico público e privado) do Concelho de Coimbra, levando-se em consideração a Carta Educativa do Município de Coimbra 2008/2015. Utilizaram-se na comparação diversos indicadores genéricos, demográficos e sociais. Caso houvesse apenas uma escola EB1 em uma freguesia, essa era escolhida obrigatoriamente, no sentido de obter-se uma representatividade das freguesias do Concelho de Coimbra. Para além dessa questão, foram levados em consideração também diversos aspectos, tais como a seleção de escolas de maior dimensão, sua envolvência, sua atividade humana, o tráfego de automóveis nas proximidades da escola e a atividade industrial desenvolvida nas respectivas zonas. No que diz respeito ao método de seleção da amostra, optou-se por uma amostra do tipo não probabilística de conveniência. Os critérios de inclusão foram os seguintes: seleção de ao menos uma escola por freguesia, utilização dos critérios de comparação enumerados anteriormente e autorização da parte da Direção Regional de Educação do Centro (DREC), dos agrupamentos de escolas e dos diretores dessas para a participação no estudo. A amostra das instituições foi constituída por 51 escolas, correspondendo a 81 salas de aula (35, 34 e 12 salas do 1º ano, do 4º ano e mista, respectivamente). Do total das escolas, 32 encontravam-se localizadas em freguesias de tipologia predominantemente urbana, 17 localizavam-se em freguesias de tipologia medianamente urbana, e 2 localizavam-se em freguesias predominantemente rurais.(11)

Foi avaliada a qualidade do ar interno das salas de aula incluídas no estudo em duas ocasiões, nas estações outono/inverno e primavera/verão. Para caracterizar a qualidade do ar interno foram avaliadas a temperatura, a umidade relativa do ar (UR), assim como concentrações de monóxido de carbono (CO), dióxido de carbono (CO_2_), ozônio (O_3_), dióxido de nitrogênio (NO_2_), dióxido de enxofre (SO_2_), compostos orgânicos voláteis (COVs), formaldeído, material particulado com diâmetro de 2,5 µm (MP_2,5_) e MP com diâmetro de 10 µm (MP_10_). As avaliações foram realizadas no outono/inverno (entre novembro de 2010 e fevereiro de 2011) e na primavera/verão (entre março de 2011 e junho de 2011).

A norma técnica governamental NT-SCE-02(12) menciona que as medições dos poluentes devem ser efetuadas no período representativo de ocupação, passadas entre duas a três horas após o início da atividade dos espaços ou quando tenham sido obtidas as condições de equilíbrio. As medições da qualidade do ar interno ocorreram no período normal de funcionamento das aulas, aproximadamente duas horas após o começo das aulas (da manhã ou da tarde), colocando-se o equipamento na posição mais central de cada sala e, sensivelmente, à altura das vias respiratórias dos alunos quando sentados. As medições foram efetuadas de acordo com o estabelecido pela norma técnica governamental,(12) a uma altura de 1 m do solo e a uma distância mínima de 3 m das paredes entre 10:30 e 17:30, durante um período de 30 min, com amostragens para MP a cada 30 s, para COVs a cada 15 s, e para os restantes parâmetros a cada 60 s. Foram realizadas essas medições durante uma semana. Dependendo das dimensões das salas de aula, fizeram-se, em média, duas medições por dia em cada sala.

As avaliações da qualidade do ar ambiente externo ocorreram no espaço de recreio dos alunos, à mesma altura a que foram efetuadas as medições da qualidade do ar interno, mas afastada ao menos 1 m das paredes exteriores das escolas em estudo.(13)

Utilizaram-se equipamentos portáteis específicos de leitura em tempo real: VelociCalc 9555-P (TSI Inc., Shoreview, MN, EUA), para a medição de temperatura, UR, e concentrações de CO e CO_2_; monitor série 500 (Aeroqual Ltd., Auckland, Nova Zelândia), para a medição da concentração de O_3_; QRAE (ERA Systems Europe ApS, Kastrup, Dinamarca) para a medição da concentração do NO_2_ e SO_2_; Formaldemeter htV (PPM Technology, Caernarfon, Reino Unido), para a avaliação de formaldeído; Voyager (Photovac Inc., Waltham, MA, EUA), para a medição da concentração de COVs; e DUSTTRACK (TSI Inc.); para a medição da concentração de MP. Os equipamentos foram calibrados antes de qualquer período de amostragem, recorrendo-se sempre que necessário ao "branco" ou padrão-zero, com base comparativa dos resultados encontrados nos casos de medidas com troca de sensores. Foi levada em consideração a conversão das leituras de acordo com as variações de temperatura e pressão.

As informações sobre os alunos foram recolhidas por um questionário que resultou de diferentes momentos de pré-teste. Esses momentos focaram no tempo de preenchimento por parte das famílias (pais e encarregados de educação) e compreensão das perguntas consoante as temáticas. A versão final, de forma genérica, debruçou-se sobre as seguintes áreas temáticas: características da família (nuclear, unipessoal ou alargada), características habitacionais (local de residência, tempo médio de permanência, tipo de habitação, condições térmicas, etc.) e informações relativas a sintomas/patologias e nível de atividade física das crianças. Os questionários foram entregues pelo investigador por carta (envelope) aos professores de cada sala, e esses a fizeram chegar através dos alunos aos seus pais/encarregados.

A seleção dos alunos do EB1 teve por base um tipo de amostragem não probabilístico e acidental/conveniência (técnica de amostragem). De um universo de 4.319 crianças do EB1, foram selecionadas 1.019.

Foram realizadas medições de peso e altura das crianças e, a partir dessas, foi calculado o índice de massa corpórea, dividindo-se o peso (kg) pelo quadrado da estatura (m). As crianças quando apresentavam esse índice acima do percentil 95 foram classificadas como com excesso de peso, utilizando-se a distribuição em percentis para sexo e idade elaborada pelo *Centers for Disease Control and Prevention* em 2000.(14)

A descrição dos resultados foi feita por escola, e as proporções foram comparadas pelo teste de qui-quadrado e teste exato de Fisher. Para a comparação das variáveis contínuas foi usada ANOVA ou seu equivalente não paramétrico, o teste de Kruskal-Wallis. Quanto à estimativa de risco relativo, essa foi estimada através de OR e respectivos IC95%. Utilizamos o programa IBM SPSS Statistics, versão 19.0 (IBM Corporation, Armonk, NY, EUA), para as análises.

No presente estudo, optamos estrategicamente por classificar o nível de CO_2_ das instituições escolares em duas categorias: ausência de risco, caso o valor médio obtido fosse inferior à concentração máxima de referência de acordo com o Decreto-Lei no. 79/2006 do governo português ( ≤ 984 ppm); e presença de risco, caso os valores obtidos atingissem valores > 984 ppm.

Resultados

As médias de idade dos alunos que frequentavam o 1º e 4º anos foram de 6,20 ± 0,42 anos e 9,25 ± 0,48 anos, respectivamente. A maioria das crianças era do sexo masculino (51,63%). Observou-se, nas 493 crianças do sexo feminino, uma distribuição relativamente equilibrada nos dois anos estudados. Uma tendência semelhante foi observada nos alunos do sexo masculino.

Ao medirmos o peso e a altura, verificamos que 5% das crianças em estudo eram obesas.

Verificou-se ainda, que 84,6% das crianças praticavam esportes. Constatamos também que, em 7 escolas, 100% das crianças praticavam esportes.

Ao descrevermos o nível educacional dos pais/responsáveis por área de residência (freguesias), verificamos que, dos 1.014 respondentes, 436 (43%) tinham formação superior, e 48 (4,7%) desses somente tinha o EB1. Também constatamos que a maioria dos pais residia em freguesias predominantemente urbanas e medianamente urbanas (61% e 26%, respectivamente).

Relativamente à idade das habitações onde as crianças residiam, foi possível verificar que houve diferenças estatisticamente significativas: 71,3% das habitações tinham menos de 21 anos, e 8,2% tinham mais de 40 anos. Além disso, 25,4% das residências apresentavam bolores, e houve diferenças significativas em relação à presença de umidade nas habitações e à presença de sistema de aquecimento domiciliar (sua falta afetava 53,7% das crianças).

Foi possível ainda verificar que as escolas nas quais a escolaridade dos pais/responsáveis era mais elevada havia uma maior proporção de alunos com aquecimento nas suas habitações e com menos sinais de umidade em casa.

Os valores da concentração média dos parâmetros analíticos ambientais nos espaços internos nas diferentes instituições de ensino nos dois momentos de amostragem (outono/inverno e primavera/verão) estão descritos na Tabela 1.

**Tabela 1:** Distribuição dos valores da concentração de poluentes no ar ambiente interno das 81 salas de aula estudadas em função da estação do ano.a

Poluentes	Período de amostragem		Δoutono/inverno – primavera/verão (média)	Concentração máxima de referência na legislação portuguesa
	Outono/inverno	Primavera/verão		
CO, ppm	0,42 ± 0,53*	0,14 ± 0,13	0,28	10,7
CO_2_, ppm	1578,16* ± 12,49	1152,80 ± 95,41	425,36	984
MP2,5, mg/m^3^	0,08 ± 0,04	0,10 ± 0,03	−0,02	Não há
MP10, mg/m^3^	0,12 ± 0,05	0,11 ± 0,03	0,006	0,15
O_3_, ppm	0,002 ± 0,060	0,0009 ± 0,0040	0,001	0,10
COVs, ppb	97,82 ± 73,72	90,51 ± 65,66	7,31	260
SO_2_, ppm	0,005 ± 0,020	0,004 ± 0,030	0,001	Não há
Formaldeído, ppm	0,01 ± 0,01*	0,02 ± 0,02	−0,01	0,08

CO : monóxido de carbono

CO2 : dióxido de carbono

MP2,5 : material particulado com diâmetro de 2,5 μm

MP10 : MP com diâmetro de 10μm

O3 : ozônio

COVs : compostos orgânicos voláteis

SO2 : dióxido de enxofre

a Valores expressos em média ± dp

* p < 0,0001 (teste t de Student para amostras pareadas ou teste T de Wilcoxon).

Como podemos observar, foi na amostragem durante o outono/inverno que se registraram valores médios de CO e CO_2_ significativamente superiores (p < 0,001) face à amostragem durante primavera/verão nos mesmos espaços de ensino. No que diz respeito ao parâmetro CO, a redução entre as duas fases de avaliação foi de 0,28 ppm. Ao nível do parâmetro analítico CO_2_, a redução desse parâmetro no período primavera/verão foi de 425,36 ppm. Devemos realçar que a concentração máxima de referência de CO_2_ (984 ppm) foi largamente ultrapassada, ou seja, os alunos nesses ambientes estão em presença de risco para a saúde.

Em sentido inverso, registraram-se alterações médias estatisticamente significativas entre a amostragem durante o outono/inverno face àquela durante a primavera/verão no que diz respeito ao parâmetro analítico formaldeído. A amostragem de primavera/verão revelou estimativas médias significativamente superiores de formaldeído em comparação com aquela de outono/inverno. O aumento nos níveis de formaldeído foi de 0,0103 ppm. Os restantes parâmetros, apesar de não apresentarem alterações estatisticamente significativas entre os dois momentos de avaliação, indicaram que os parâmetros MP_10_, O_3_, COVs e SO_2_ apresentaram uma redução na amostragem de primavera/verão em relação à de outono/inverno. Em sentido inverso, houve um aumento não significativo de MP_2,5_.

Verificamos também que, na amostragem durante o outono/inverno, as temperaturas médias do ar analisadas em todas as salas de aula, exceto em 2 escolas, estavam muito abaixo do valor de referência. Verificamos que, de uma forma geral, durante a primavera/verão, os valores das temperaturas do ar estavam acima do valor referenciado devido à temperatura exterior e ao fato das salas não possuírem sistema de arrefecimento.

Verificamos que, nos dois períodos de amostragem, os valores de UR estavam compreendidos entre os limites inferior e o superior (30-70%). No entanto, 7 escolas, no período outono/inverno, apresentaram valores de UR acima dos 70%.

Quando controlamos os poluentes atmosféricos nos espaços externos das diferentes escolas em estudo por estação do ano, constatamos que os parâmetros atmosféricos CO, CO_2_, MP_2,5_, MP_10_ e formaldeído revelaram uma variação média significativa. Em relação aos níveis de CO e CO_2_, esses revelaram uma redução significativa durante o outono/inverno em relação à primavera/verão. Em sentido inverso, registrou-se um agravamento dos níveis de MP_2,5_, MP_10_ e formaldeído durante a primavera/verão em relação ao outono/inverno.

Na Tabela 2 podemos observar a sintomatologia e as patologias mais prevalentes nas crianças estudadas.

**Tabela 2:** Sinais, sintomas e patologias nas crianças do primeiro e quarto anos no primeiro ciclo do ensino básico, Coimbra, Portugal.

Sinais, sintomas e patologias		Ano de escolaridade						Total	
		1º ano			4º ano				
		n	% coluna	% linha	n	% coluna	% linha	n	% coluna
Asma	Não	451	89,3	50,2	448	87,2	49,8	899	88,2
	Sim	54	10,7	45,0	66	12,8	55,0	120	11,8
	Total	505	100,0	49,6	514	100,0	50,4	1.019	100,0
Bronquite crônica	Não	495	98,0	49,6	502	97,7	50,4	997	97,8
	Sim	10	2,0	45,5	12	2,3	54,5	22	2,2
	Total	505	100,0	49,6	514	100,0	50,4	1.019	100,0
Estertores/sibilos	Não	420	83,2	48,6	444	86,4	51,4	864	84,8
	Sim	85	16,8	54,8	70	13,6	45,2	155	15,2
	Total	505	100,0	49,6	514	100,0	50,4	1.019	100,0
Crises de espirros	Não	382	75,6	50,3	377	73,3	49,7	759	74,5
	Sim	123	24,4	47,3	137	26,7	52,7	260	25,5
	Total	505	100,0	49,6	514	100,0	50,4	1.019	100,0
Rinite alérgica	Não	426	84,4	51,0	409	79,6	49,0	835	81,9
	Sim	79	15,6	42,9	105	20,4	57,1	184	18,1
	Total	505	100,0	49,6	514	100,0	50,4	1019	100,0
Dificuldades respiratórias	Não	459	90,9	50,1	457	88,9	49,9	916	89,9
	Sim	46	9,1	44,7	57	11,1	55,3	103	10,1
	Total	505	100,0	49,6	514	100,0	50,4	1.019	100,0
Estresse	Não	499	98,8	49,9	502	97,7	50,1	1001	98,2
	Sim	6	1,2	33,3	12	2,3	66,7	18	1,8
	Total	505	100,0	49,6	514	100,0	50,4	1.019	100,0
Tonturas	Não	497	98,4	49,7	502	97,7	50,3	999	98,0
	Sim	8	1,6	40,0	12	2,3	60,0	20	2,0
	Total	505	100,0	49,6	514	100,0	50,4	1019	100,0
Irritabilidade	Não	487	96,4	49,9	489	95,1	50,1	976	95,8
	Sim	18	3,6	41,9	25	4,9	58,1	43	4,2
	Total	505	100,0	49,6	514	100,0	50,4	1.019	100,0
Dores de cabeça	Não	472	93,5	50,4	465	90,5	49,6	937	92,0
	Sim	33	6,5	40,2	49	9,5	59,8	82	8,0
	Total	505	100,0	49,6	514	100,0	50,4	1019	100,0
Irritação mucosas olhos	Não	483	95,6	49,8	486	94,6	50,2	969	95,1
	Sim	22	4,4	44,0	28	5,4	56,0	50	4,9
	Total	505	100,0	49,6	514	100,0	50,4	1.019	100,0
Insônia	Não	486	96,2	49,7	492	95,7	50,3	978	96,0
	Sim	19	3,8	46,3	22	4,3	53,7	41	4,0
	Total	505	100,0	49,6	514	100,0	50,4	1019	100,0
Tosse	Não	421	83,4	49,2	434	84,4	50,8	855	83,9
	Sim	84	16,6	51,2	80	15,6	48,8	164	16,1
	Total	505	100,0	49,6	514	100,0	50,4	1.019	100,0
Falta de concentração	Não	402	79,6	50,8	390	75,9	49,2	792	77,7
	Sim	103	20,4	45,4	124	24,1	54,6	227	22,3
	Total	505	100,0	49,6	514	100,0	50,4	1.019	100,0

Os sintomas/patologias mais prevalentes que as crianças do 1º ano do EB1 apresentaram foram: crise de espirros, em 24%; falta de concentração, em 20%; estertores e sibilos, em 17%; tosse, em 16%; e rinite alérgica, em 16%. Quanto às crianças que frequentavam o 4º ano do EB1, os sintomas/patologias mais prevalentes foram: crise de espirros, em 27%, falta de concentração, em 24%; rinite alérgica, em 20%; e tosse, em 16%. Quando comparamos a presença de cada sintoma em função do ano de escolaridade frequentado, constatamos que as crianças do 1º ano face àquelas do 4º ano apresentavam mais frequentemente estertores e sibilos, em 55%; e tosse, em 51%. Os restantes sintomas/patologias comparados revelaram ocorrer com maior frequência nas crianças que se encontravam no 4º ano do EB1.

Entre os parâmetros ambientais analisados, o nível de CO_2_ revelou os piores resultados, com elevado risco potencial. As concentrações médias de CO_2_ no interior das salas de aula encontravam-se, de uma forma geral, bastante acima da concentração máxima de referência (984 ppm), chegando a valores de até 1.942 ppm. Visto que as concentrações de CO_2_ encontradas nas salas de aula no outono/inverno apresentaram valores muito superiores às concentrações verificadas na primavera/verão, procuramos estimar o risco de sintomas/patologias das crianças expostas naquelas estações. As salas de aula foram categorizadas como com "presença de risco" ou "ausência de risco" em relação ao valor de referência. Os sintomas/patologias foram relatados pelos pais/responsáveis através do questionário (Tabela 3).

**Tabela 3:** Estimativa de risco de sintomas e patologias respiratórias das crianças expostas à concentração média superior ou inferior à concentração máxima de referência de dióxido de carbono durante o período de amostragem outono/inverno.

Sintomas e patologias respiratórias	Concentração máxima de referência de dióxido de carbono							c2	gl	p	OR	IC95%
	Superior			Inferior		Total						
	n		%	n	%	n	%					
Asma	Sim	100	11,7	20	12,3	120	11,8	0,046	1	0,831	0,946	0,567-1,579
	Não	756	88,3	143	87,7	899	88,2					
	Total	856	100,0	163	100,0	1019	100,0					
Bronquite crônica	Sim	18	2,1	4	2,5	22	2,2	0,080	1	0,777	0,854	0,285-2,556
	Não	838	97,9	159	97,5	997	97,8					
	Total	856	100,0	163	100,0	1019	100,0					
Estertores/sibilos	Sim	133	15,5	22	13,5	155	15,2	0,442	1	0,506	1,179	0,725-1,917
	Não	723	84,5	141	86,5	864	84,8					
	Total	856	100,0	163	100,0	1019	100,0					
Crise de espirros	Sim	223	26,1	37	22,7	260	25,5	0,810	1	0,368	1,200	0,807-1,784
	Não	633	73,9	126	77,3	759	74,5					
	Total	856	100,0	163	100,0	1019	100,0					
Rinite alérgica	Sim	160	18,7	24	14,7	184	18,1	1,457	1	0,227	1,331	0,835-2,122
	Não	696	81,3	139	85,3	835	81,9					
	Total	856	100,0	163	100,0	1019	100,0					
Tosse	Sim	141	16,5	23	14,1	164	16,1	0,565	1	0,452	1,200	0,745-1,933
	Não	715	83,5	140	85,9	855	83,9					
	Total	856	100,0	163	100,0	1019	100,0					
Dificuldades respiratórias	Sim	85	9,9	18	11,0	103	10,1	0,187	1	0,666	0,888	0,518-1,522
	Não	771	90,1	145	89,0	916	89,9					
	Total	856	100,0	163	100,0	1019	100,0					

gl : graus de liberdade.

Não se verificou uma associação significativa entre a presença/ausência de asma e a exposição a espaços de salas com presença/ausência de risco (p = 0,831). No entanto, a prevalência de asma foi de 11,8% no total da população de crianças em estudo.

A bronquite crônica ocorreu em 22 crianças (2,2%); porém, não se verificou uma associação significativa entre a presença dessa patologia e a exposição a valores de CO_2_ elevados nas salas de aula durante o outono/inverno (p > 0,05). Os sintomas estertores/sibilos foram relatados em 155 crianças (prevalência de 15,2%), e tampouco houve uma associação significativa (p > 0,05). Apesar de não se observar uma associação entre crise de espirros e exposição a valores de CO_2_ elevados (p > 0,05), verificamos que das 856 crianças expostas a salas com níveis de CO_2_ elevado (presença de risco), 223 (26,1%) sofriam desse sintoma.

No que diz respeito à rinite alérgica, tosse e dificuldades respiratórias, não houve associações significativas entre esses sintomas e exposição a salas com presença e ausência de risco face ao poluente atmosférico CO_2_ (p > 0,05); no entanto, 184 crianças (18,9%) sofriam de rinite, 164 (16,1%) apresentavam tosse, e 103 (10,1%) tinham dificuldades respiratórias face à totalidade das crianças controladas em função de cada sintoma.

Procuramos compreender a distribuição de sintomas não respiratórios em função das salas de aula classificadas como com presença ou ausência de risco durante a estação outono/inverno. A classificação de presença de risco e ausência de risco seguiu a mesma metodologia apresentada na análise anterior (Tabela 4).

**Tabela 4:** Estimativa de risco de sinais e sintomas não respiratórios das crianças expostas à concentração média inferior ou superior à concentração máxima de referência de dióxido de carbono durante o período de amostragem outono/inverno.

Sinais e sintomas	Concentração máxima de referência de dióxido de carbono							c2	gl	p	OR	IC95%
	Superior			Inferior		Total						
	n		%	n	%	n	%					
Estresse	Sim	14	1,6	4	2,5	18	1,8	0,529	1	0,467	0,661	0,215-2,034
	Não	842	98,4	159	97,5	1001	98,2					
	Total	856	100,0	163	100,0	1019	100,0					
Tonturas	Sim	17	2,0	3	1,8	20	2,0	0,015	1	0,902	1,081	0,313-3,730
	Não	839	98,0	160	98,2	999	98,0					
	Total	856	100,0	163	100,0	1019	100,0					
Irritabilidade	Sim	40	4,7	3	1,8	43	4,2	2,718	1	0,099	2,614	0,799-8,554
	Não	816	95,3	160	98,2	976	95,8					
	Total	856	100,0	163	100,0	1019	100,0					
Dores de cabeça	Sim	70	8,2	12	7,4	82	8,0	0,123	1	0,726	1,121	0,593-2,118
	Não	786	91,8	151	92,6	937	92,0					
	Total	856	100,0	163	100,0	1019	100,0					
Irritação das mucosas	Sim	42	4,9	8	4,9	50	4,9	0,0001	1	0,999	1,000	0,460-2,171
	Não	814	95,1	155	95,1	969	95,1					
	Total	856	100,0	163	100,0	1019	100,0					
Insônia	Sim	33	3,9	8	4,9	41	4,0	0,393	1	0,531	0,777	0,352-1,714
	Não	823	96,1	155	95,1	978	96,0					
	Total	856	100,0	163	100,0	1019	100,0					
Falta de concentração	Sim	206	24,1	21	12,9	227	22,3	9,888	1	0,002	2,143	1,320-3,478
	Não	650	75,9	142	87,1	792	77,7					
	Total	856	100,0	163	100,0	1019	100,0					

gl : graus de liberdade.

A partir do relato dos pais/responsáveis das 1.019 crianças do estudo, pudemos calcular a prevalência dos seguintes sinais e sintomas: estresse, 1,8%; tonturas, 2,0%; irritabilidade, 4,2%; dores de cabeça, 8%; irritação das mucosas, 4,9%; e insônia, 4,0%. Não houve associações significativas entre nenhum dos parâmetros avaliados e os tipos de riscos nos espaços de aula (p > 0,05). Em relação ao sinal "falta de concentração", constatou-se que essa estava associada a crianças expostas a ar interno nas salas de aula com valores de CO_2_ > 984 ppm (p = 0,002). A probabilidade de estar exposto a valores de CO_2_ > 984 ppm e apresentar falta de concentração foi 2,143 vezes maior do que nas crianças não expostas a essas concentrações. A prevalência desse sinal ocorreu em 227 crianças (22,3%) face à totalidade das crianças em estudo.

Procuramos verificar se a presença de asma nas crianças poderia estar relacionada com a exposição domiciliar ao fumo de tabaco (Tabela 5). Constatamos que 361 pais/responsáveis (35,43%) eram fumantes e, desses, 252 (69,8%) tinham por hábito fumar em casa. Não se verificou uma associação entre a exposição ao tabaco e a presença de asma (p > 0,05); no entanto, das 252 crianças expostas ao fumo de tabaco, 30 (11,9%) apresentavam asma.

**Tabela 5:** Distribuição de pais/responsáveis fumantes (N = 361) que fazem uso domiciliar de tabaco e sua relação com asma nas crianças.

Uso domiciliar de tabaco	Asma na criança			p	OR	IC95%
	Sim	Não	TOTAL			
Sim	30 (11,9)	222 (88,1)	252 (100,0)	0,305	1,502	0,687-3,280
Não	9 (8,3)	100 (91,7)	109 (100,0)			
TOTAL	39 (10,8)	322 (89,2)	361 (100,0)			

## Discussão

As crianças são um grupo de risco e podem apresentar vulnerabilidades quando expostas a um ambiente interior de menor qualidade. O desenvolvimento de patologias respiratórias está associado à fraca qualidade do ar em edifícios escolares.^(^ ^6^ ^,^ ^15^ ^)^

No presente estudo, as concentrações dos poluentes monitorizados estavam em geral abaixo da concentração máxima de referência, exceto a de CO_2_. No entanto, alguns dos parâmetros apresentavam valores significativos, nomeadamente COVs e MP_10_.

Constatou-se face aos resultados obtidos que existe uma fraca renovação de ar no interior das salas de aulas. A volumetria das salas, o número de ocupantes das salas e as condições climáticas no exterior não possibilitam que o arejamento das salas durante os intervalos seja suficiente para diminuir os níveis de CO_2_ a valores aceitáveis. Vários estudos recentes, alguns portugueses,^(^ ^2^ ^,^ ^16^ ^,^ ^17^ ^)^ têm revelado a existência de teores de CO_2_ elevados em escolas devido à elevada densidade de ocupação e à insuficiente ventilação.^(^ ^18^ ^-^ ^21^ ^)^ Tais resultados levantam uma série de questões que deverão ser alvo de reflexão por parte dos governantes e de quem tem responsabilidades nessa área, em especial após as últimas reestruturações efetuadas ao nível das escolas e dos agrupamentos. Ao serem criados mega agrupamentos, aumentando, consequentemente, o número de alunos por sala, o número de turmas irá diminuir, o que nos leva a colocar a seguinte questão: Não estaremos a diminuir a qualidade do ar interno e, consequentemente, a diminuir a saúde das crianças?

No presente estudo, os sintomas/patologias mais prevalentes foram as crises de espirros, seguidas pela falta de concentração, rinite alérgica, tosse, estertores/sibilos e asma. Outros estudos referem resultados semelhantes a esses.^(^ ^6^ ^,^ ^22^ ^,^ ^23^ ^)^ Verificou-se ainda, que a falta de concentração revelou estar associada ao ar interno das salas de aula com valores de CO_2_ > 984 ppm (p = 0,002). A probabilidade de crianças que estão expostas a valores acima dos níveis de referência para CO_2_ e ter falta de concentração foi 2,143 vezes maior do que nas crianças não expostas. Em um estudo, valores elevados de CO_2_ em escolas associaram-se a ocorrência de estertores e tosse em crianças.^(^ ^24^ ^)^

A exposição ao fumo de tabaco em ambientes internos resulta em um aumento dos riscos para a bronquite, asma, entre outros.^(^ ^25^ ^,^ ^26^ ^)^ Dado termos verificado que, dos 361 pais/responsáveis fumantes, 30,2% não fumavam em casa e 69,8% fumavam na habitação, procuramos avaliar a relação existente entre os pais que fumavam dentro da habitação e a sintomatologia/patologia das crianças e verificamos que dos pais/responsáveis que fumavam dentro da habitação, a maioria desses tinham crianças com asma (76,9%), bronquite crônica, estertores/sibilos (69,0%), crise de espirros (56,0%), rinite alérgica (65,0%), estresse (66,7%), tonturas (85,7%), irritabilidade (71,4%), dores de cabeça (75,0%), irritação das mucosas dos olhos (66,7%), tosse seca (53%), insônia (72,7%), dificuldades respiratórias (70,5%) e falta de concentração (62,2%). Os efeitos para a saúde da exposição passiva ao fumo do tabaco têm sido alvo de inúmeras investigações. Sabe-se que as crianças são particularmente suscetíveis, com um risco aumentado de desenvolvimento de doença alérgica das vias aéreas, designadamente de asma brônquica, e há intensificação da gravidade da mesma.^(^ ^27^ ^,^ ^28^ ^)^ Nesse sentido, e face aos nossos resultados, que são preocupantes do ponto de vista ambiental nas escolas, seria desejável que as crianças estivessem expostas a valores inferiores de todos os contaminantes em casa, incluindo os contaminantes da exposição ao fumo do tabaco.

Hoje em dia, as pessoas passam a maior parte do tempo em locais fechados, como a escola. A fraca qualidade do ar nessas edificações associa-se ao desenvolvimento de patologias respiratórias. No presente estudo, o sintoma com maior prevalência foi crises de espirros, seguido por falta de concentração.

A maioria das escolas estudadas tinha uma qualidade do ar e conforto térmico razoáveis, embora a concentração de vários poluentes, sobretudo CO_2_, possa indicar a oportunidade de se efetuar intervenções corretivas, reduzindo as fontes emissoras e promovendo maior ventilação. Vários estudos têm revelado a existência de teores de CO_2_ elevados em escolas, causados quer pela elevada densidade de ocupação, quer pela insuficiente ventilação.^(^ ^2^ ^,^ ^18^ ^-^ ^21^ ^)^ Verificamos uma associação estatisticamente significativa entre apresentar falta de concentração e estar exposto a valores elevados de CO_2_. Uma possível explicação para a falta de significância estatística para as outras variáveis estudadas é o baixo nível de poluição na cidade de Coimbra.

Como potenciais limitações do estudo, podemos considerar as informações referentes aos sintomas/patologias dos alunos, dado essas terem sido relatadas pelos pais/responsáveis. A percepção que pais/responsáveis têm em relação a suas crianças poderia eventualmente não corresponder à realidade.

O presente estudo permitiu avaliar os riscos a que a população está exposta e fornecer diretrizes para a elaboração de medidas que visem a minimização desses riscos. Espera-se que os resultados encontrados contribuam para o planejamento da saúde ambiental em edifícios escolares e para o aperfeiçoamento de estratégias políticas promotoras de qualidade de vida.
